# Resolving lipoxin A_4_: Endogenous mediator or exogenous anti-inflammatory agent?

**DOI:** 10.1016/j.jlr.2024.100734

**Published:** 2024-12-24

**Authors:** Reagan M. McGuffee, Matthew A. Luetzen, David A. Ford

**Affiliations:** Edward A. Doisy Department of Biochemistry and Molecular Biology and Center for Cardiovascular Research, Saint Louis University School of Medicine, St. Louis, MO, USA

Specialized proresolving mediators (SPMs) are an enzymatically produced subset of oxylipins. SPMs have primarily inflammation-resolving actions including promotion of neutrophil clearance, regulation of cytokine production, and analgesic properties (1). The SPMs, protectins, resolvins, and maresins, originate from the omega-3 PUFA species EPA and DHA. In contrast, lipoxins, which were the first class of SPMs to be described roughly 40 years ago, originate from the omega-6 PUFA arachidonic acid (AA) (2). The two major lipoxins, lipoxin A_4_ and lipoxin B_4_, are proposed to be synthesized in two enzymatic oxygenation steps mediated by 5-lipoxygenase activity, which is enhanced by 5-lipoxygenase-activating protein, followed by either 12-lipoxygenase or 15-lipoxygenase activity (3). Neutrophils, macrophages, and platelets are enzymatically competent to produce lipoxins. The lipoxin A_4_ receptor (ALX) had been proposed to be the G protein-coupled receptor formylmethionyl-leucyl-phenylalanine receptor 2 (FPR2), also known as the lipoxin A_4_ receptor (4). This receptor is expressed on several immune cells and is believed to be the major receptor activated by lipoxins to mediate neutrophil chemotaxis and macrophage efferocytosis among other immune modulatory actions (5).

Questions have been raised, particularly over the last decade, concerning the validity of the analytical methods used to detect and quantify SPMs and whether any observed SPM anti-inflammatory effects are truly the result of FPR2 activation. A recent review by O’Donnell *et al.* (6) highlighted suspect methods employed to detect resolvins and maresins by LC-MS/MS in biological samples described in a study by Gomez *et al.* (7). Among the problematic criteria for detection and quantification were *1*) not following signal-to-noise (S/N) ratio standards for detection and quantification and *2*) disproportionate abundances and incomplete overlap of product ions between analytical standards and the reported natural SPMs in samples. Upon closer inspection, the detection of many SPMs in the Gomez study was below an acceptable S/N ratio of 5:1 for quantitation and some even below 3:1, introducing doubt about their presence in the samples. To ensure accuracy of results, limits of detection and quantification (LOD and LOQ) are primarily determined utilizing the S/N ratios and fragmentation patterns. Regarding LOD and LOQ ratios, accepted limits are 3:1 (S/N) for detection and at least 5:1 (S/N) for quantification utilizing MS data without smoothing (6). In addition, in review of the previous literature detecting and quantifying SPMs, O’Donnell *et al.* (6) found that MS studies did not adhere to accepted guidelines set for LOD and LOQ as well as had problems with minimum counts per second and alignment of retention times. While SPM affinities for the FPR2 receptor are reported to be quite high with an approximate *K*_d_ of 1 nM for lipoxin A_4_ (8) and 0.2 nM for resolvin D_1_ (9), in vivo and ex vivo reports of SPM concentrations remain concerning since they are frequently of low picomolar concentrations (10, 11, 12). A separate review by Schebb *et al.* (13) also outlined the pitfalls of enzyme-linked immunosorbent assays, a common and convenient method for clinical detection of SPMs, which often overestimate SPM levels likely because of low analyte specificity among the multitude of similarly structured SPMs.

The study by Gelhaus *et al.* has further evaluated the role of lipoxin A_4_ in biological systems. Immortalized murine macrophages (RAW 264.7 cells) and C57BL6/J mouse bone marrow-derived macrophages were treated with lipopolysaccharide (LPS) as well as precursor AA to examine lipoxin A_4_ production in these systems. Lipoxin A_4_ was not detected in both cell lysate and media from LPS-stimulated cells using an LC-MS technique with robust sensitivity at 30 pg of lipoxin A_4_-d_5_ standard. The authors also confirmed by Western blot the expression of 5-lipoxygenase, 12-lipoxygenase, 15-lipoxygenase, and 5-lipoxygenase-activating protein expression in these cells. As anticipated, prostaglandins and HETEs were formed and detected following LPS activation. In addition, exogenously added lipoxin A_4_ was shown to be metabolized to 15-oxo-lipoxin A_4_, which was likely mediated by 15-hydroxyprostaglandin dehydrogenase. Under LPS stimulation and AA supplementation, natural production of this 15-oxo-lipoxin A_4_ was likewise not observed in these macrophages.

Further investigations by Gelhaus *et al.* focused on the role of the FPR2 receptor for exogenous lipoxin A_4_ signaling. FPR2 was expressed under all conditions in RAW 264.7 cells. High nanomolar levels of lipoxin A_4_ and 15-oxo-lipoxin A_4_, which is well above most reported in vivo concentrations and above the reported *K*_d_ of FPR2 for lipoxin A_4_, were incapable of initiating GTPγS binding to G_i_. This indicated no activation of FPR2 by classical G protein-coupled receptor agonist binding. Conversely, WKYMVm, a hexapeptide FPR2 agonist, was sufficient to induce FPR2 activation by this assay. A clever alternative for lipoxin A_4_ anti-inflammatory signaling was then explored, focusing on the electrophilic nature of the 15-oxo-lipoxin A_4_. Some studies have reported α,β unsaturated carbonyl oxylipin products of 15-hydroxyprostaglandin dehydrogenase can have anti-inflammatory activity through the NF-κB pathway, which is likely mediated by cysteine modification of NF-κB factors by Michael addition of the electrophilic oxylipin metabolites (14, 15, 16). The Gelhaus group validated the electrophilic properties of 15-oxo-lipoxin A_4_ in vitro demonstrating the production of a glutathione conjugate, which was detected by LC–MS/MS in this in vitro experiment as well as in RAW 264.7 cells treated with an esterified 15-oxo-lipoxin A_4_. While assessing NF-κB activation and nuclear erythroid 2-related factor 2 (Nrf2) in a follow-up treatment of RAW 264.7 cells with lipoxin A_4_ and 15-oxo-lipoxin A_4_, it was revealed that 15-oxo-lipoxin A_4_ alone could significantly decrease LPS-induced cytokine secretion and trigger gene and protein expression of Nrf2 protective factors associated with glutathione production and pro-oxidant detoxification (Figure 1).

**Fig. 1:**
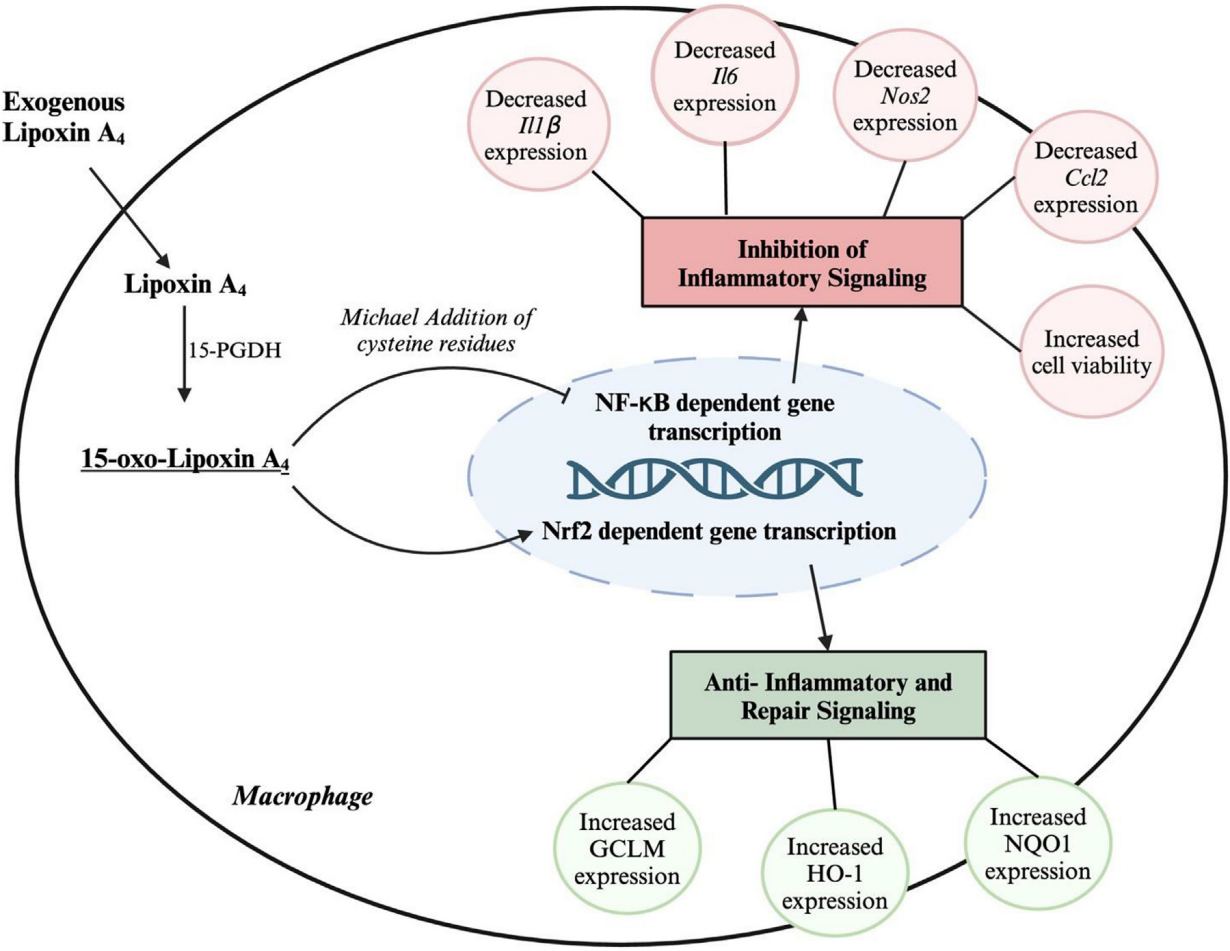
Anti-inflammatory mechanisms of exogenous lipoxin A_4_(LXA_4_). Exogenous LXA_4_ enters the macrophage where it is oxidized by 15-hydroxyprostaglandin dehydrogenase (15-PGDH) yielding 15-oxo-lipoxin A_4_. 15-Oxo-lipoxin A_4_ is an electrophilic α, β-unsaturated ketone metabolite capable of inducing a signal response through post-translational modification of redox-sensitive proteins. Gelhaus *et al.* found that two protein signaling pathways, NF-ĸB and Nrf2, were altered by 15-oxo-lipoxin A_4_ during the inflammatory response. The modification of NF-ĸB by 15-oxo-lipoxin A_4_ is likely through Michael addition at cysteine residues and results in the inhibition of the NF-ĸB-dependent gene transcription. These effects were reported in the downregulation of interleukin-1β (*IL1β*), interleukin-6 (*IL6*), monocyte chemoattractant protein 1 (*Ccl2*), and inducible nitric oxide synthase 2 (*Nos2*) resulting in decreased proinflammatory signaling. Additionally, 15-oxo-lipoxin A_4_ activated Nrf2-dependent anti-inflammatory gene transcription, resulting in increased expression of glutamate-cysteine ligase modifier subunit (GCLM), heme oxygenase 1 (HO-1), and NAD(P)H quinone oxidoreductase 1 (NQO1). Given the electrophilic nature of 15-oxo-lipoxin A_4_, it likely targets other redox-sensitive signaling pathways. Created with BioRender.com.

Lipoxin A_4_ has potential therapeutic value, albeit the studies by Gelhaus *et al.* indicate it may not be an endogenously produced anti-inflammatory lipid. The mechanism of action now is likely through an NF-kB-Nrf2 signaling pathway mediated by the lipoxin A_4_ catabolite 15-oxo-lipoxin A_4_ rather than through direct lipoxin A_4_ agonist activity with FPR2. Importantly, the studies by Gelhaus *et al.* reinforced the need for careful scrutiny of LC-resolved peaks in respect to S/N, precise retention times, and compound fragmentation patterns.

## Conflict of interest

The authors declare that they have no conflicts of interest with the contents of this article.

## References

[1] Serhan C.N., Dalli J., Colas R.A., Winkler J.W., Chiang N. (2015). Protectins and maresins: new pro-resolving families of mediators in acute inflammation and resolution bioactive metabolome. Biochim. Biophys. Acta.

[2] Serhan C.N., Hamberg M., Samuelsson B. (1984). Trihydroxytetraenes: a novel series of compounds formed from arachidonic acid in human leukocytes. Biochem. Biophys. Res. Commun..

[3] Chandrasekharan J.A., Sharma-Walia N. (2015). Lipoxins: nature's way to resolve inflammation. J. Inflamm. Res..

[4] Serhan C.N., Levy B.D., Clish C.B., Gronert K., Chiang N. (2000). Lipoxins, aspirin-triggered 15-epi-lipoxin stable analogs and their receptors in antiinflammation: a window for therapeutic opportunity. Ernst Schering Res. Found Workshop.

[5] Serhan C.N., Chiang N., Van Dyke T.E. (2008). Resolving inflammation: dual anti-inflammatory and pro-resolution lipid mediators. Nat. Rev. Immunol..

[6] O'Donnell V.B., Schebb N.H., Milne G.L., Murphy M.P., Thomas C.P., Steinhilber D. (2023). Failure to apply standard limit-of-detection or limit-of-quantitation criteria to specialized pro-resolving mediator analysis incorrectly characterizes their presence in biological samples. Nat. Commun..

[7] Gomez E.A., Colas R.A., Souza P.R., Hands R., Lewis M.J., Bessant C. (2020). Blood pro-resolving mediators are linked with synovial pathology and are predictive of DMARD responsiveness in rheumatoid arthritis. Nat. Commun..

[8] Fiore S., Maddox J.F., Perez H.D., Serhan C.N. (1994). Identification of a human cDNA encoding a functional high affinity lipoxin A4 receptor. J. Exp. Med..

[9] Krishnamoorthy S., Recchiuti A., Chiang N., Yacoubian S., Lee C.H., Yang R. (2010). Resolvin D1 binds human phagocytes with evidence for proresolving receptors. Proc. Natl. Acad. Sci. U. S. A..

[10] Calder P.C. (2020). Eicosapentaenoic and docosahexaenoic acid derived specialised pro-resolving mediators: concentrations in humans and the effects of age, sex, disease and increased omega-3 fatty acid intake. Biochimie.

[11] Skarke C., Alamuddin N., Lawson J.A., Li X., Ferguson J.F., Reilly M.P., FitzGerald G.A. (2015). Bioactive products formed in humans from fish oils. J. Lipid Res..

[12] Ebert R., Cumbana R., Lehmann C., Kutzner L., Toewe A., Ferreirós N. (2020). Long-term stimulation of toll-like receptor-2 and -4 upregulates 5-LO and 15-LO-2 expression thereby inducing a lipid mediator shift in human monocyte-derived macrophages. Biochim. Biophys. Acta Mol. Cell Biol. Lipids.

[13] Schebb N.H., Kühn H., Kahnt A.S., Rund K.M., O’Donnell V.B., Flamand N. (2022). Formation, signaling and occurrence of specialized pro-resolving lipid mediators-what is the evidence so far?. Front. Pharmacol..

[14] Wei C., Zhu P., Shah S.J., Blair I.A. (2009). 15-oxo-Eicosatetraenoic acid, a metabolite of macrophage 15-hydroxyprostaglandin dehydrogenase that inhibits endothelial cell proliferation. Mol. Pharmacol..

[15] Snyder N.W., Revello S.D., Liu X., Zhang S., Blair I.A. (2013). Cellular uptake and antiproliferative effects of 11-oxo-eicosatetraenoic acid. J. Lipid Res..

[16] Hee S.W., Chang Y.-C., Su L., Chen I.-J., Jeng Y.-M., Hsieh M.-L. (2023). 15-keto-PGE2 alleviates nonalcoholic steatohepatitis through its covalent modification of NF-κB factors. iScience.

